# Mitogen-Activated Protein Kinase 15 Is a New Predictive Biomarker and Potential Therapeutic Target for Ovarian Cancer

**DOI:** 10.3390/ijms25010109

**Published:** 2023-12-20

**Authors:** Qiu-Hua Zhong, Andy T. Y. Lau, Yan-Ming Xu

**Affiliations:** Laboratory of Cancer Biology and Epigenetics, Department of Cell Biology and Genetics, Shantou University Medical College, Shantou 515041, China; 17qhzhong@stu.edu.cn

**Keywords:** MAPK15, biomarker, therapeutic target, ovarian cancer

## Abstract

Mitogen-activated protein kinase 15 (MAPK15) has been reported to be associated with several cancers. This study aimed to explore for the first time on the relationship between MAPK15 expression and cancer progression/drug responsiveness in ovarian carcinoma. To this end, MAPK15 expression level was examined by immunohistochemistry (IHC) staining of an ovarian tissue array (10 normal and 70 malignant samples). Drug sensitivity of ovarian cancer cell lines (including OVCAR3 and SKOV3) was measured by MTS assay. The modulation of MAPK15 expression in OVCAR3 and SKOV3 was verified by immunoblot and real-time PCR analyses. The prognostic value of MAPK15 in ovarian cancer patients was assessed using the Kaplan-Meier Plotter database and Gene Expression Omnibus (GEO) datasets. The IHC results showed that MAPK15 expression was negatively associated with tumor grade, TNM stage, tumor size, and regional lymph node metastasis of ovarian carcinoma. Importantly, overexpressing MAPK15 increased cisplatin toxicity in ovarian carcinoma cells and online database analysis indicated that patients with high MAPK15 expression had favorable prognosis with/without chemotherapy. Taken together, our results indicate that a decreased MAPK15 expression is associated with advanced-stage ovarian cancer and unfavorable survival outcomes. MAPK15 may be a new biomarker for ovarian cancer, and the encouraging therapeutic strategy would be found by combining the regulation of MAPK15 expression.

## 1. Introduction

Ovarian cancer is the leading cause of mortality among patients suffering gynecological malignancies, for which the 5-year survival rate is about 40% [1,2]. As there are no obvious symptoms at the early stage, ovarian cancer patients are usually diagnosed with this disease at the advanced stage. The primary treatment for ovarian cancer is cytoreductive surgery combined with adjuvant therapy, which generally consists of platinum/paclitaxel-based chemotherapy [3]. Predictive biomarkers are being investigated to improve targeted therapies for ovarian cancer, which include anti-angiogenic drugs, PARP inhibitors, folate receptor antibody-drug conjugates, as well as immune checkpoint inhibitors [4]. The aberrant activation of the mitogen-activated protein kinase (MAPK) pathway has been reported for ovarian cancer, with elevated MAPK activities enabling the resistance to platinum, paclitaxel, or PARP inhibitors [5].

MAPK15 (ERK8), the most recently discovered atypical MAPK, was identified through a library screen using the ERK7 cDNA probe [6]. Recent studies show that MAPK15 is involved in tumorigenesis and the progression of several cancers through regulating tumor development, distant metastasis, and drug susceptibility. MAPK15 contributes to carcinogenesis, and it is activated by RET/PTC3 and RET/MEN2B, which are constitutively active forms of the RET proto-oncogene [7]. Using high-throughput functional RNA interference (RNAi), Cerone et al. revealed that MAPK15 is a novel regulator of telomerase, whose inhibition reduces telomerase activity and elicits characteristics of telomere dysfunction in cancer cells [8]. Interestingly, MAPK15 is a chromatin-bound kinase, which protects genomic integrity by controlling the proliferating cell nuclear antigen (PCNA) levels in normal cycling mammary cells [9]. MAPK15 has also been reported to stimulate autophagy by interacting with L3 and GABARAP proteins in the tumorigenic process [10]. In addition, MAPK15 copy number gains are observed in premalignant gastric lesions, and may promote malignant transformation by affecting c-Jun phosphorylation and stability, as well as cell cycling [11]. Likewise, MAPK15 is highly expressed in the malignant components of male germ cell tumor, and its upregulation may contribute to the malignant transformation of germ cells by controlling a “stress support” autophagic pathway, which protects the cells from the accumulation of DNA damage, and consequently prevents the activation of tumor suppressor TP53 and TP53-mediated cell cycle arrest [12]. Our previous research revealed that MAPK15 promotes metastasis via its interaction with NF-κB subunit p50 and the transcriptional regulation of prostaglandin E2 receptor EP3 subtype in human non-small cell lung cancer (NSCLC) [13]. Moreover, our group has reported the pivotal role of MAPK15 and NF-κB signaling in boosting the efficacy of combination therapy with cisplatin and TNF-α, as well as increasing arsenic trioxide-induced cell apoptosis, in lung cancer cell lines [14,15]. However, the expression and regulation of MAPK15 are still unexplored in many other types of cancer. In the present study, for the first time, we characterize the relationship of MAPK15 with clinicopathological parameters and prognostic outcomes in ovarian cancer. We found that MAPK15 is a pivotal factor of carcinogenesis and drug responsiveness, therefore influencing patient survival.

## 2. Results

### 2.1. MAPK15 Expression in Ovarian Tissues

**Figure 1:**
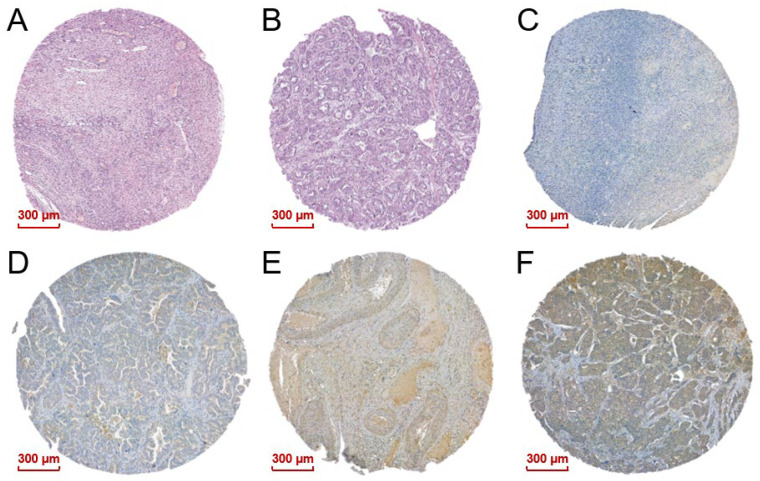
Representative photomicrographs of ovarian TMA sections. H&E-stained TMA section: (**A**) Normal tissue; (**B**) malignant tissue. IHC staining: (**C**–**F**) MAPK15-stained photomicrographs of (**C**) negative, (**D**) weak, (**E**) moderate, and (**F**) strong staining.

In order to investigate the MAPK15 expression level in ovarian carcinoma, we performed IHC staining with MAPK15 antibody. MAPK15 was distributed mainly in the cytoplasm of tumor cells, but was expressed in the nucleus as well. Representative examples of the hematoxylin & eosin (H&E) and IHC staining results of ovarian tissue microarray sections are shown in Figure 1. In the tissue microarray, among the 70 malignant ovarian samples, 64.3% (45 samples) were defined as having a low expression of MAPK15, and 35.7% (25 samples) had high expression levels, while in 10 normal ovarian samples, 90% (nine samples) had low expression, and 10% (one sample) had high expression (Table 1).

### 2.2. Association of MAPK15 Expression with Clinicopathological Parameters in the Tissue Microarray Analysis

**Figure 2:**
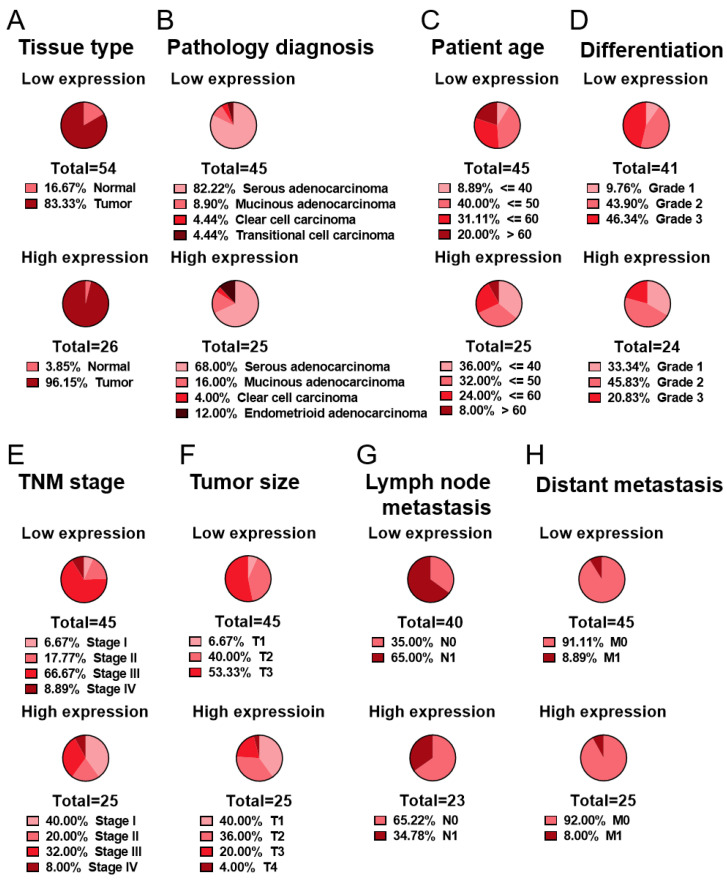
Frequency distribution of clinicopathological factors grouped by MAPK15 expression, in relation to Table 1 and Table 2.

Then, the ovarian tissue samples were grouped based on clinicopathological factors, which were then correlated with MAPK15 expression. MAPK15 expression was negatively correlated with tumor grade (*p* = 0.0243), tumor size (*p* = 0.0236), regional lymph node metastasis (*p* = 0.0348), and tumor stage (*p* = 0.0046) of ovarian cancer (Table 2). These relationships can be visualized as pie charts. The low-expression groups comprised a higher ratio of elderly patients, poorly differentiated tumors, and advanced stage (Figure 2C–E), suggesting that MAPK15 expression is involved in the carcinogenesis and progression of ovarian cancer. Moreover, a larger tumor size and a greater extent of lymph node metastasis, which are two parameters of the tumor stage, also constituted a higher fraction in the low-expression groups compared with the high-expression groups (Figure 2F,G). These correlations suggest that MAPK15 participates in ovarian cancer progression though affecting tumor growth and metastasis.

### 2.3. MAPK15 Expression Increases Cisplatin Sensitivity in Ovarian Carcinoma Cell Lines

**Figure 3:**
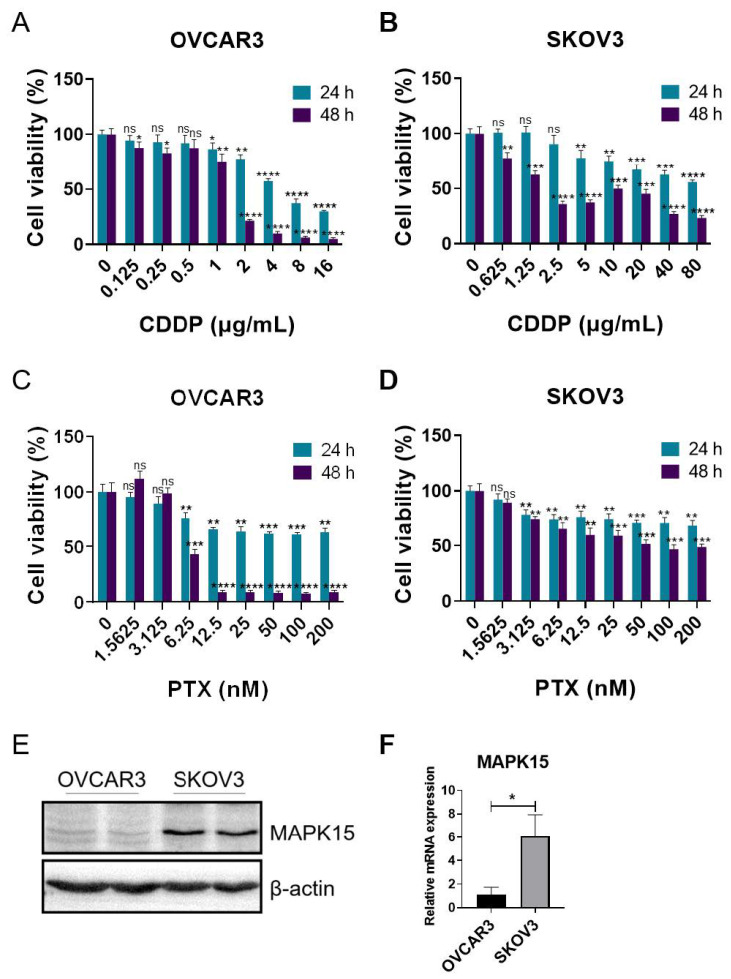
Drug sensitivity and MAPK15 expression in ovarian cell lines. (**A**,**B**) OVCAR3 and SKOV3 cells were treated with different concentrations of cisplatin (CDDP). (**C**,**D**) OVCAR3 and SKOV3 cells were treated with different concentrations of paclitaxel (PTX). (**E**) MAPK15 protein expression in OVCAR3 and SKOV3 cells. The protein samples were loaded in duplicate. (**F**) MAPK15 mRNA expression in OVCAR3 and SKOV3 cells. ns, *p* > 0.05; *, *p* < 0.05; **, *p* < 0.01; ***, *p* < 0.001; ****, *p* < 0.0001.

**Figure 4:**
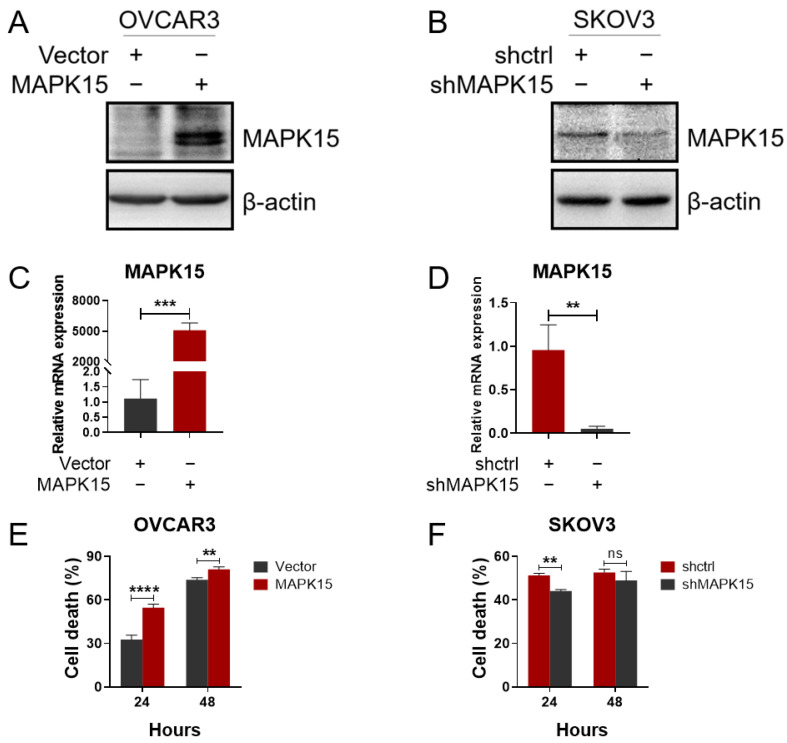
Drug sensitivity in ovarian cell lines following engineered alterations in MAPK15 expression level. (**A**,**C**) MAPK15 overexpression in pcDNA4/HisMaxA-MAPK15-transfected OVCAR3 cells. (**B**,**D**) MAPK15 knockdown in pLKO.1-TRC-shMAPK15-transfected SKOV3 cells. (**E**) MAPK15 overexpression increased OVCAR3 cell death under 2.5 μg/mL cisplatin treatment. (**F**) MAPK15 knockdown decreased the SKOV3 cell death under 25 μg/mL cisplatin treatment. ns, *p* > 0.05; **, *p* < 0.01; ***, *p* < 0.001; ****, *p* < 0.0001.

To further investigate whether MAPK15 also has an effect on cancer chemotherapy, we measured the chemotherapy susceptibility of ovarian cell lines with altered MAPK15 expression. First, we determined the sensitivity of the OVCAR3 and SKOV3 cell lines to cytotoxic drugs. In both OVCAR3 and SKOV3 cells, cisplatin treatment induced cell death in a dose-dependent manner (Figure 3A,B), whereas paclitaxel-induced cell death did not follow a concentration-dependent manner (Figure 3C,D). Since OVCAR3 cells displayed a relatively lower MAPK15 expression at the protein and mRNA levels, in comparison with SKOV3 cells (Figure 3E,F); therefore, we overexpressed and knocked down MAPK15 in OVCAR3 and SKOV3 cells respectively, and verified the changes in MAPK15 expression by immunoblot and qPCR analyses (Figure 4A–D). The transfected ovarian cancer cell lines were subcultured in 96-well plates and treated with cisplatin. Notably, in OVCAR3 cells, MAPK15 overexpression conferred greater sensitivity to cisplatin treatment (Figure 4E). Conversely, in SKOV3 cells, the knockdown of MAPK15 conferred resistance to cisplatin treatment (Figure 4F).

### 2.4. Patients with High MAPK15 Expression Have Favorable Prognosis

**Figure 5:**
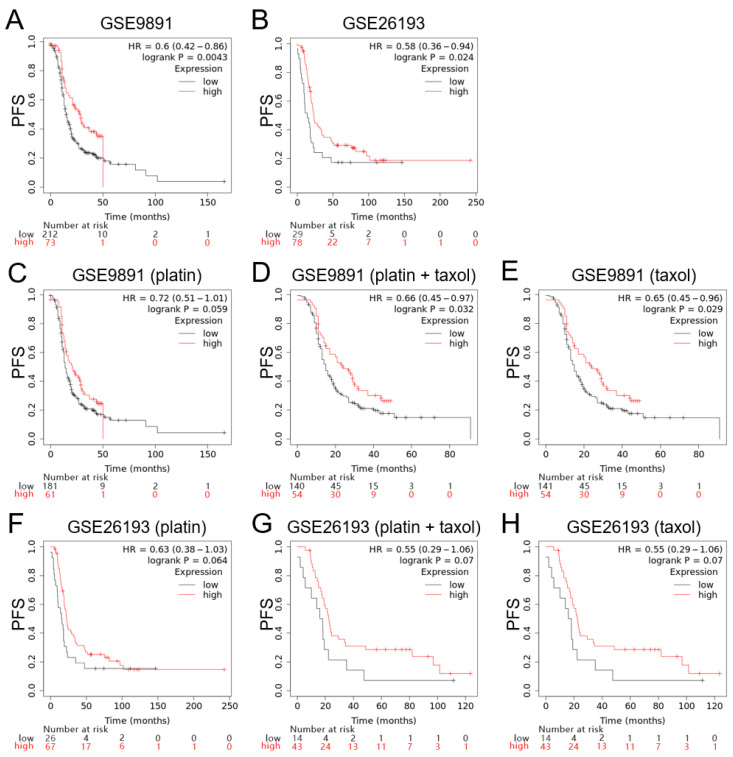
Kaplan-Meier curves based on MAPK15 expression showing progression-free survival (PFS) of patients with ovarian carcinoma. (**A**) PFS according to GSE9891. (**B**) PFS according to GSE26193. (**C**–**E**) PFS according to GSE9891 with platin and/or taxol chemotherapy. (**F**–**H**) PFS according to GSE26193 with platin and/or taxol chemotherapy.

Since MAPK15 clearly impacts ovarian cancer development and chemotherapy, we proposed a hypothesis that MAPK15 expression could be an indicator of ovarian cancer patient survival. Here, the survival analyses were performed using Kaplan–Meier Plotter (https://kmplot.com/ accessed on 14 October 2022) and GEO datasets. We chose the 241357_at as the MAPK15 probe to perform the analyses based on GSE9891 and GSE26193 datasets. The progression-free survival (PFS) for patients with high MAPK15 expression was longer than for patients with low MAPK15 expression (GSE9891: HR = 0.6, 95% CI = 0.42–0.86, *p* = 0.0043; GSE26193: HR = 0.58, 95% CI = 0.36–0.94, *p* = 0.024) (Figure 5A,B). The overall survival (OS) curves showed the same tendency as PFS, but did not reach significance (GSE9891: HR = 0.74, 95% CI = 0.47–1.18, *p* = 0.21; GSE26193: HR = 0.64, 95% CI = 0.39–1.06, *p* = 0.082) (Supplementary Figure S3A,B).

### 2.5. Patients with Chemotherapy in the High-MAPK15 Expression Group Have Favorable Prognosis

Furthermore, we introduced chemotherapy into the survival analysis. When treated with platin and/or taxol, a high MAPK15 expression level indicated favorable outcomes in GSE9891 dataset (platin: HR = 0.72, 95% CI = 0.51–1.01, *p* = 0.059; platin + taxol: HR = 0.66, 95% CI = 0.45–0.97, *p* = 0.032; taxol: HR = 0.65, 95% CI = 0.45–0.96, *p* = 0.029) (Figure 5C–E). Consistently, survival analyses based on GSE26193 dataset showed similar results (Figure 5F–H).

These findings suggest that MAPK15 expression is a beneficial factor for ovarian cancer patients through enhancing the efficacy of platinum/paclitaxel-based chemotherapy.

## 3. Discussion

Ovarian cancer accounts for the most lethal malignant disease among all gynecological carcinomas. Although the diagnosis and therapeutic strategy have been constantly improving, the 5-year survival rate of ovarian cancer patients is still less than 40%, while that for breast cancer is 85% [4], and is due to the latent and nonspecific symptoms at the early stage, which hindered timely diagnosis and resulted in rapid recurrence. Since the treatment window is short, effective and diverse treatment options are particularly important. Predictive biomarkers offer promise for the evolution of precision medicine, which can contribute to more precise therapeutic strategies [4]. In our study, we demonstrate MAPK15 is a novel biomarker capable of predicting the outcome of ovarian cancer, and as a contributor to cisplatin-related chemotherapy.

MAPK15 is a newly identified MAPK that can be activated through starvation-serum stimulation, DNA damage, hydrogen hydroxide, arsenic trioxide, cigarette smoke extract, and human oncogenes, such as RET/PTC3 (an active form of proto-oncogene RET) [16,17].

MAPK15, which contains two SH3-binding motifs in its C-terminal region, associates with the c-Src SH3 domain in vitro and co-immunoprecipitates with c-Src in vivo. The fact that transfection with either v-Src or a constitutively active c-Src increases MAPK15 activation indicates that MAPK15 can be one of the downstream targets of the Src-dependent signaling pathway [6].

The signaling pathway of MAPK15 plays critical roles in many cellular responses. In 2012, Colecchia et al. reported the novel function of MAPK15 in autophagy. A conserved LC3-interacting region (LIR) from position 340 to 343 in MAPK15 was suggested to be responsible for its interaction with ATG8 family proteins (MAP1LC3B, GABARAP, and GABARAPL1) and its localization to autophagic structures, consequently, the stimulation of the formation of these compartments [10]. In addition, the same research group demonstrated BCR-ABL-induced autophagy modulated by MAPK15 through its ability to interact with LC3-family proteins in an LIR-dependent manner. Afterward, MAPK15 regulates oncogene-dependent cell proliferation and the tumor formation of human chronic myeloid leukemia via physically recruiting the oncogene to autophagic vesicles [18]. Particularly, it was well investigated that MAPK15 directly interacts with the ULK1 complex and localizes onto the autophagosomes to control early phases of the autophagic process induced by starvation [10,19]. MAPK15-ULK1 signaling is upregulated in the airway epithelia of chronic obstructive pulmonary disease (COPD) patients. In the human airway epithelial cell line, cigarette smoke extract activates MAPK15-ULK1 signaling through increasing the phosphorylation of MAPK15, ULK1 (Ser555), the expression of Beclin1, and the ratio of LC3II/LC3I, therefore exacerbating mitophagy and mitochondrial oxidative stress [17].

As a chromatin-bound kinase, MAPK15, protects genomic integrity by inhibiting the E3 ubiquitin-protein HDM2-mediated degradation of the DNA clamp PCNA. MAPK15 contains a conserved PCNA-interacting protein (PIP) box and MAPK15 controls PCNA levels by preventing its destruction via HDM2 in normal cycling mammary epithelial cells [9].

Our previous study sheds light for the first time on MAPK15′s role as an oncokinase, whereby the epidermal growth factor-activated MAPK15 phosphorylates the proto-oncogene c-Jun at Ser 63/73 and increases AP-1 activity, resulting in an enhanced anchorage-independent cell transformation and tumorigenesis of colon cancer [20]. Then, other researchers revealed that MAPK15 in gastric cancer is associated with copy number gain and contributes to the malignant transformation by prolonging the stability of c-Jun [11]. Moreover, MAPK15 is indicated to be an osteosarcoma metastasis-associated gene, and is confirmed to promote the proliferation, migration, and invasion by activating the c-Jun/MMP signaling pathways [21].

Recently, our group has reported that MAPK15-mediated NF-κB activation enhances the arsenic trioxide-induced apoptosis through promoting the phosphorylation and degradation of IκBα, as well as the nuclear translocation of NF-κB in lung cancer cells [15]. Accordingly, we have demonstrated that the transcriptional upregulation of MAPK15 via NF-κB signaling boosts the efficacy of TNF-α-augmented cisplatin-induced cell apoptosis [14]. More recently, we clarify that MAPK15 interacts with NF-κB p50 and enters the nucleus, in which NF-κB p50 binds to the EP3 promoter and transcriptionally regulates the expression of EP3, thereby affecting the migration of lung adenocarcinoma [13].

Furthermore, aberrant MAPK15 expression has been reported in various cancers, such as breast cancer [22], lung neuroendocrine neoplasms [23], and gastric cancers [11]. In breast cancer, MAPK15 may play a role in maintaining tissue homeostasis through regulating the stability and activity of ERα, while the loss of MAPK15 expression is closely related to breast cancer progression [22,24]. Here, we found a deficiency of MAPK15 expression in poorly differentiated ovarian carcinomas (Figure 2D; Supplementary Figure S1D), which suggests that MAPK15 plays a pivotal role in the carcinogenesis of ovarian cancer. However, MAPK15 IHC staining in normal tissue is relatively weak when compared with the malignant tissue (Supplementary Figure S1A,B). Combined with existing research showing that MAPK15 could boost DNA damage [14] and stimulate autophagy [10], we propose that when cells encounter hits, MAPK15 is upregulated, as a stress-activated protein, to enhance the DNA damage and cell death, which subsequently prevents abnormal cell accumulation and malignant transformation. On the other hand, those cells that lack MAPK15 upregulation are headed for a disastrous outcome―carcinogenesis.

MAPK15 works as a key player of GalNAc-transferase relocalization to regulate cell migratory ability, and the loss of MAPK15 could make cancer cells more aggressive [25]. Our previous study showed that MAPK15 is required for distant metastasis in lung adenocarcinoma through regulating EP3 [13]. MAPK15 deficiency is also involved in advanced stages through increasing tumor size and lymph node metastasis (Figure 2E–G; Supplementary Figure S1E–G) in ovarian cancer, which suggests that MAPK15 closely participates in tumor development.

Noteworthy, our results demonstrate that MAPK15 expression could enhance cisplatin cytotoxicity in ovarian cancer cells. Overexpressing MAPK15 can dramatically increase cisplatin-induced cell death in cells with low MAPK15 expression, but is less effective in high MAPK15-expressing cells (Supplementary Figure S2). Consistently, survival analyses indicated MAPK15 is a favorable factor for the prognosis of ovarian cancer patients. Hence, upregulating MAPK15 expression will potentially personalize therapeutic strategies for patients with low MAPK15 levels.

In conclusion, we have found that MAPK15 expression prolongs ovarian cancer patient survival through regulating cancer progression and enhancing chemotherapy effectiveness. MAPK15 deficiency is associated with advanced-stage ovarian cancer and unfavorable survival outcomes. It would be valuable to further investigate the molecular mechanism of MAPK15 regulation in ovarian carcinoma.

## 4. Materials and Methods

### 4.1. Tissue Microarray

A tissue microarray was purchased from Avilabio (Biomax, Rockville, MD, USA), in which the samples were fixed in 10% formalin within 15–30 min after surgery for 24 h to 48 h, then processed in a Leica tissue processor and embedded in paraffin. Each slide was sectioned, producing 4 μm sections, and the diameter of each core was 1.5 mm. The tissue microarray (OV809) we used in the study included 80 cases of ovarian tissue with various clinical diagnoses. The patient information and tissue clinicopathological parameters are provided in Supplementary Table S1.

### 4.2. Immunohistochemistry Assay

For immunohistochemistry (IHC), the tissue microarray section was deparaffinized with xylene after heating at 60 °C for 10 min, hydrated with gradient concentrations of ethanol (100%, 10 min; 95%, 90%, 70%, 0%, 5 min, respectively), blocked at room temperature by incubating in 3% H_2_O_2_ for 40 min to quench the endogenous hydrogen peroxidase. Then, heat-induced epitope retrieval was performed using 0.01 mol/L citrate buffer (pH 6.0) for 20 min in a water bath at 98 °C. After allowing to cool at room temperature, sections were blocked with 5% BSA for 30 min at room temperature to prevent non-specific adsorption. Subsequently, the sections were incubated with anti-MAPK15 polyclonal antibody (1:100) at 4 °C overnight, and then incubated with an HRP-conjugated secondary antibody (1:500) at room temperature for 45 min. Immunoreactions were visualized using a 3,3-diaminobenzidine tetrahydrochloride (DAB) substrate kit (Zhongshan Golden Bridge Inc., Beijing, China) for 5 min [26]. After counterstaining with hematoxylin, the array was scanned using a digital microscope scanning platform for subsequent evaluation and analysis.

### 4.3. Evaluation of Immunohistochemical Staining

For analysis of MAPK15 expression, we used the well-established immunoreactivity scoring system comprising qualitative and quantitative dimensions [27]. The intensity category of immunostaining was qualitatively graded as follows: 0, no staining; 1, weak staining; 2, moderate staining; 3, strong staining. The percentage category of immunostaining was quantitatively measured as follows: 0, 0–5%; 1, 6–25%; 2, 26–50%; 3, 51–75%; 4, 76–100%. The immunoreactivity scoring system was calculated by multiplying the scores of the two dimensions. According to the immunoreactivity scoring system, low expression was defined as a total score of 0–8, and high expression was defined as a total score of 9–12.

### 4.4. Cell Culture and Transfection

The OVCAR3 and SKOV3 ovarian cancer cell lines were purchased from the National Collection of Authenticated Cell Cultures. OVCAR3 cells were cultured in ATCC-modified RPMI 1640 medium (30-2001) supplemented with 20% fetal bovine serum (Gibco, Grand Island, NY, USA) and 1% penicillin–streptomycin (Gibco, Grand Island, NY, USA). SKOV3 cells were cultured in McCoy’s 5A medium (Sigma-Aldrich, St. Louis, MO, USA) supplemented with 10% fetal bovine serum and 1% penicillin–streptomycin. The cells were maintained in a 5% CO_2_-humidified incubator at 37 °C. Transfections were performed with PEI reagent according to the manufacturer’s instructions. The plasmids pcDNA4/HisMaxA-MAPK15 and pLKO.1-TRC-shMAPK15 have been described previously [14].

### 4.5. Immunoblot Analysis

After washing 3 times with ice-cold PBS, cells were scraped into a 1.5 mL tube. Cell pellets were collected after centrifugation for 5 min at 1000× *g*, 4 °C. Then, cells were lysed in radio-immunoprecipitation assay (RIPA) buffer and sonicated. The cell lysates were then centrifuged at 16,900× *g* for 10 min at 4 °C and the supernatants collected for protein quantitation. Equal amounts of protein were used for immunoblot analysis as described previously [28]. Membranes were blocked with 5% skimmed milk in PBST followed by the incubation with anti-MAPK15 polyclonal antibody (1:500) at 4 °C overnight. After washing with PBST, membranes were incubated with HRP-conjugated secondary antibody at room temperature for 2 h. Finally, protein signals were visualized with the Enhanced Chemiluminescence Detection Kit (GE Healthcare, Uppsala, Sweden) under a Chemiluminescence Imaging System (Tanon 5200, Shanghai, China). Gray scales were analyzed using Gel-Pro Analyzer.

### 4.6. Real-Time PCR

Total cellular RNA was extracted with TRIzol reagent (Invitrogen, Carlsbad, CA, USA), and cDNA was synthesized using a GoScript Reverse Transcription System (Promega, A5001, Madison, WI, USA) following the manufacturer’s instructions. Real-time PCR was performed using GoTaq qPCR Master Mix (Promega, A6001, Madison, WI, USA) on an Applied Biosystems QS5 Real-Time system, with specific primers for MAPK15 (forward: 5′-GACCAGAAGCCGTCCAATGT-3′; reverse: 5′-GTATCGGTGCGAAGAGAGCA-3′) and β-actin (forward: 5′-GAGCTACGAGCTGCCTGACG-3′; reverse: 5′-CTCCATGCCCAGGAAGGAAGG-3′). Primers were synthesized by IGE Biotechnology, Ltd. (Guangzhou, China). Relative mRNA expression was calculated by the 2^−ΔΔCT^ method [29] and normalized to β-actin (internal control).

### 4.7. Cell Viability Assay

Cells were seeded into 96-well plates (OVCAR3: 50,000 cells/well; SKOV3: 15,000 cells/well). At 24 h after seeding, cells were treated with various concentrations of cisplatin or paclitaxel for 48 h. At 0, 24, and 48 h after cisplatin or paclitaxel addition, MTS/PMS (Promega, Madison, WI, USA) was added, and cells were further incubated for 1.5 h. Then, 100 μL of cell medium was transferred to empty 96-well plates and measured at 492 nm wavelength.

### 4.8. Data Mining

To investigate the association between MAPK15 expression and prognosis in ovarian cancer patients, gene expression profile data were searched and downloaded from NCBI GEO (Gene Expression Omnibus). The sample screening criterion were: (1) sample type was a human ovarian tumor; (2) data type was expression profiling from array; (3) datasets had enough sample capacity and thorough clinical information; (4) platforms of the datasets included gene probes corresponding to MAPK15; and (5) patients had received chemotherapeutic treatment. According to the above criteria, the GSE9891 and GSE26193 datasets were finally chosen. The platform of the datasets was GPL570, which used the Affymetrix Human Genome U133 Plus 2.0 Array. MAPK15 was divided into a high expression group and low expression group using an optimal cut-off value.

### 4.9. Statistical Analysis

The relationship between MAPK15 protein amount and various clinicopathological characteristics was analyzed using the Fisher’s exact test. The Kaplan-Meier Plotter log-rank test was used for survival analysis. Differences with *p* < 0.05 were considered significant for the experimental data in this work.

## Figures and Tables

**Table 1:** Correlation between MAPK15 expression and ovarian tissue types.

Tissue Types	Total	MAPK15 Expression		*p*-Value
		Low (IRS 0–8)	High (IRS 9–12)	
				0.1541
Normal	10	9	1	
Malignant	70	45	25	

**Table 2:** Correlation between MAPK15 expression and clinicopathological factors in ovarian cancer samples.

Clinicopathological Parameters	Total	MAPK15 Expression		*p*-Value
		Low (IRS 0–8)	High (IRS 9–12)	
Age (years)				0.3056
≤60	59	36	23	
>60	11	9	2	
Grade				0.0243 *
1	12	4	8	
2 + 3	53	37	16	
TNM Stage				0.0046 *
I + II	26	11	15	
III + IV	44	34	10	
Tumor size				0.0236 *
T1 + T2	40	21	19	
T3 + T4	30	24	6	
Regional lymph node				0.0348 *
N0	29	14	15	
N1	34	26	8	
Distant metastasis				>0.9999
M0	64	41	23	
M1	6	4	2	

*, *p* < 0.05.

## Data Availability

All data of this study are presented in the manuscript and Supplementary Materials.

## Supplementary Materials

The following supporting information can be downloaded at: https://www.mdpi.com/article/10.3390/ijms25010109/s1.

## References

[1] Siegel R.L., Miller K.D., Fuchs H.E., Jemal A. (2022). Cancer statistics, 2022. CA Cancer J. Clin..

[2] Torre L.A., Trabert B., DeSantis C.E., Miller K.D., Samimi G., Runowicz C.D., Gaudet M.M., Jemal A., Siegel R.L. (2018). Ovarian Cancer Statistics, 2018. CA Cancer J. Clin..

[3] Armstrong D.K., Alvarez R.D., Backes F.J., Bakkum-Gamez J.N., Barroilhet L., Behbakht K., Berchuck A., Chen L.M., Chitiyo V.C., Cristea M. (2022). NCCN Guidelines(R) Insights: Ovarian Cancer, Version 3.2022. J. Natl. Compr. Canc. Netw..

[4] Lheureux S., Braunstein M., Oza A.M. (2019). Epithelial ovarian cancer: Evolution of management in the era of precision medicine. CA Cancer J. Clin..

[5] Li G.N., Zhao X.J., Wang Z., Luo M.S., Shi S.N., Yan D.M., Li H.Y., Liu J.H., Yang Y., Tan J.H. (2022). Elaiophylin triggers paraptosis and preferentially kills ovarian cancer drug-resistant cells by inducing MAPK hyperactivation. Signal Transduct. Target Ther..

[6] Abe M.K., Saelzler M.P., Espinosa R., Kahle K.T., Hershenson M.B., Le Beau M.M., Rosner M.R. (2002). ERK8, a New Member of the Mitogen-activated Protein Kinase Family. J. Biol. Chem..

[7] Iavarone C., Acunzo M., Carlomagno F., Catania A., Melillo R.M., Carlomagno S.M., Santoro M., Chiariello M. (2006). Activation of the Erk8 mitogen-activated protein (MAP) kinase by RET/PTC3, a constitutively active form of the RET proto-oncogene. J. Biol. Chem..

[8] Cerone M.A., Burgess D.J., Naceur-Lombardelli C., Lord C.J., Ashworth A. (2011). High-throughput RNAi screening reveals novel regulators of telomerase. Cancer Res..

[9] Groehler A.L., Lannigan D.A. (2010). A chromatin-bound kinase, ERK8, protects genomic integrity by inhibiting HDM2-mediated degradation of the DNA clamp PCNA. J. Cell Biol..

[10] Colecchia D., Strambi A., Sanzone S., Iavarone C., Rossi M., Dall’Armi C., Piccioni F., Verrotti di Pianella A., Chiariello M. (2012). MAPK15/ERK8 stimulates autophagy by interacting with LC3 and GABARAP proteins. Autophagy.

[11] Jin D.H., Lee J., Kim K.M., Kim S., Kim D.H., Park J. (2015). Overexpression of MAPK15 in gastric cancer is associated with copy number gain and contributes to the stability of c-Jun. Oncotarget.

[12] Rossi M., Colecchia D., Ilardi G., Acunzo M., Nigita G., Sasdelli F., Celetti A., Strambi A., Staibano S., Croce C.M. (2016). MAPK15 upregulation promotes cell proliferation and prevents DNA damage in male germ cell tumors. Oncotarget.

[13] Yu F.Y., Xu Q., Zhao X.Y., Mo H.Y., Zhong Q.H., Luo L., Lau A.T.Y., Xu Y.M. (2023). The Atypical MAP Kinase MAPK15 Is Required for Lung Adenocarcinoma Metastasis via Its Interaction with NF-κB p50 Subunit and Transcriptional Regulation of Prostaglandin E2 Receptor EP3 Subtype. Cancers.

[14] Wu D.D., Dai L.J., Tan H.W., Zhao X.Y., Wei Q.Y., Zhong Q.H., Ji Y.C., Yin X.H., Yu F.Y., Jin D.Y. (2022). Transcriptional upregulation of MAPK15 by NF-κB signaling boosts the efficacy of combination therapy with cisplatin and TNF-α. iScience.

[15] Wu D.D., Lau A.T.Y., Yu F.Y., Cai N.L., Dai L.J., Kim M.O., Jin D.Y., Xu Y.M. (2017). Extracellular signal-regulated kinase 8-mediated NF-κB activation increases sensitivity of human lung cancer cells to arsenic trioxide. Oncotarget.

[16] Lau A.T.Y., Xu Y.M. (2018). Regulation of human mitogen-activated protein kinase 15 (extracellular signal-regulated kinase 7/8) and its functions: A recent update. J. Cell. Physiol..

[17] Zhang M., Fang L., Zhou L., Molino A., Valentino M.R., Yang S., Zhang J., Li Y., Roth M. (2021). MAPK15-ULK1 signaling regulates mitophagy of airway epithelial cell in chronic obstructive pulmonary disease. Free Radic. Biol. Med..

[18] Colecchia D., Rossi M., Sasdelli F., Sanzone S., Strambi A., Chiariello M. (2015). MAPK15 mediates BCR-ABL1-induced autophagy and regulates oncogene-dependent cell proliferation and tumor formation. Autophagy.

[19] Colecchia D., Dapporto F., Tronnolone S., Salvini L., Chiariello M. (2018). MAPK15 is part of the ULK complex and controls its activity to regulate early phases of the autophagic process. J. Biol. Chem..

[20] Xu Y.M., Zhu F., Cho Y.Y., Carper A., Peng C., Zheng D., Yao K., Lau A.T.Y., Zykova T.A., Kim H.G. (2010). Extracellular signal-regulated kinase 8-mediated c-Jun phosphorylation increases tumorigenesis of human colon cancer. Cancer Res..

[21] Su Z., Yang B., Zeng Z., Zhu S., Wang C., Lei S., Jiang Y., Lin L. (2020). Metastasis-associated gene MAPK15 promotes the migration and invasion of osteosarcoma cells via the c-Jun/MMPs pathway. Oncol. Lett..

[22] Henrich L.M., Smith J.A., Kitt D., Errington T.M., Nguyen B., Traish A.M., Lannigan D.A. (2003). Extracellular signal-regulated kinase 7, a regulator of hormone-dependent estrogen receptor destruction. Mol. Cell. Biol..

[23] Motylewska E., Braun M., Kazimierczuk Z., Ławnicka H., Stępień H. (2020). IGF1R and MAPK15 Emerge as Potential Targets of Pentabromobenzylisothioureas in Lung Neuroendocrine Neoplasms. Pharmaceuticals.

[24] Rossi M., Colecchia D., Iavarone C., Strambi A., Piccioni F., Verrotti di Pianella A., Chiariello M. (2011). Extracellular signal-regulated kinase 8 (ERK8) controls estrogen-related receptor α (ERRα) cellular localization and inhibits its transcriptional activity. J. Biol. Chem..

[25] Chia J., Tham K.M., Gill D.J., Bard-Chapeau E.A., Bard F.A. (2014). ERK8 is a negative regulator of O-GalNAc glycosylation and cell migration. Elife.

[26] Cai N.L., Lau A.T.Y., Yu F.Y., Wu D.D., Dai L.J., Mo H.Y., Lin C.M., Xu Y.M. (2017). Purification and characterization of a highly specific polyclonal antibody against human extracellular signal-regulated kinase 8 and its detection in lung cancer. PLoS ONE.

[27] Remmele W., Stegner H.E. (1987). Recommendation for uniform definition of an immunoreactive score (IRS) for immunohistochemical estrogen receptor detection (ER-ICA) in breast cancer tissue. Pathologe.

[28] Wu Y.Y., Wu G.Q., Cai N.L., Xu Y.M., Lau A.T.Y. (2023). Comparison of Human Eukaryotic Translation Initiation Factors 5A1 and 5AL1: Identification of Amino Acid Residues Important for EIF5A1 Lysine 50 Hypusination and Its Protein Stability. Int. J. Mol. Sci..

[29] Livak K.J., Schmittgen T.D. (2001). Analysis of relative gene expression data using real-time quantitative PCR and the 2(-Delta Delta C(T)) Method. Methods.

