# Supplementary material for: Mitogen-Activated Protein Kinase 15 Is a New Predictive Biomarker and Potential Therapeutic Target for Ovarian Cancer
Source: Int J Mol Sci. 2023 Dec 20;25(1):109. doi: 10.3390/ijms25010109 (PMC10778700; doi:10.3390/ijms25010109)
Supplement: Supplementary file 1 [file ijms-25-00109-s001.zip › Supplementary Figures.pdf]

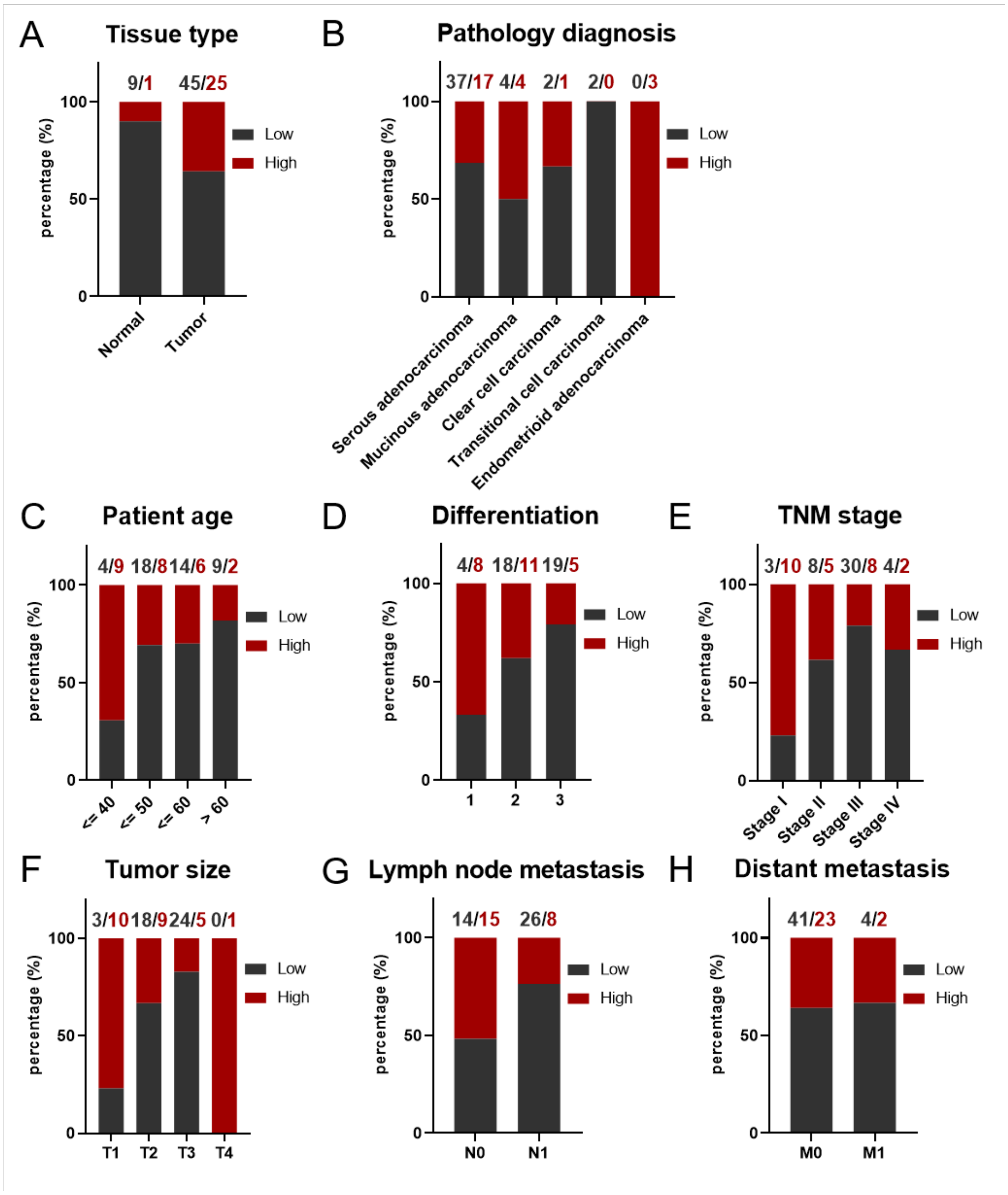

Supplementary Figure S1. Frequency distribution of MAPK15 as a function of clinicopathological factors, related to Table 1 and Table 2.

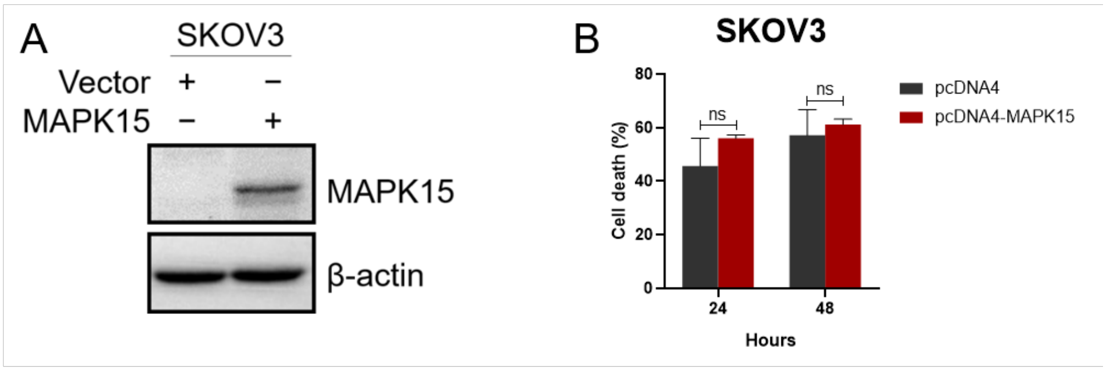

Supplementary Figure S2. Drug sensitivity in SKOV3 cells with the overexpression of MAPK15, related to Figure 4. (**A**) MAPK15 overexpression in pcDNA4/HisMaxA-MAPK15 transfected SKOV3 cells. (**B**) MAPK15 overexpression slightly increased the SKOV3 cell death under 25 μg/mL cisplatin treatment.

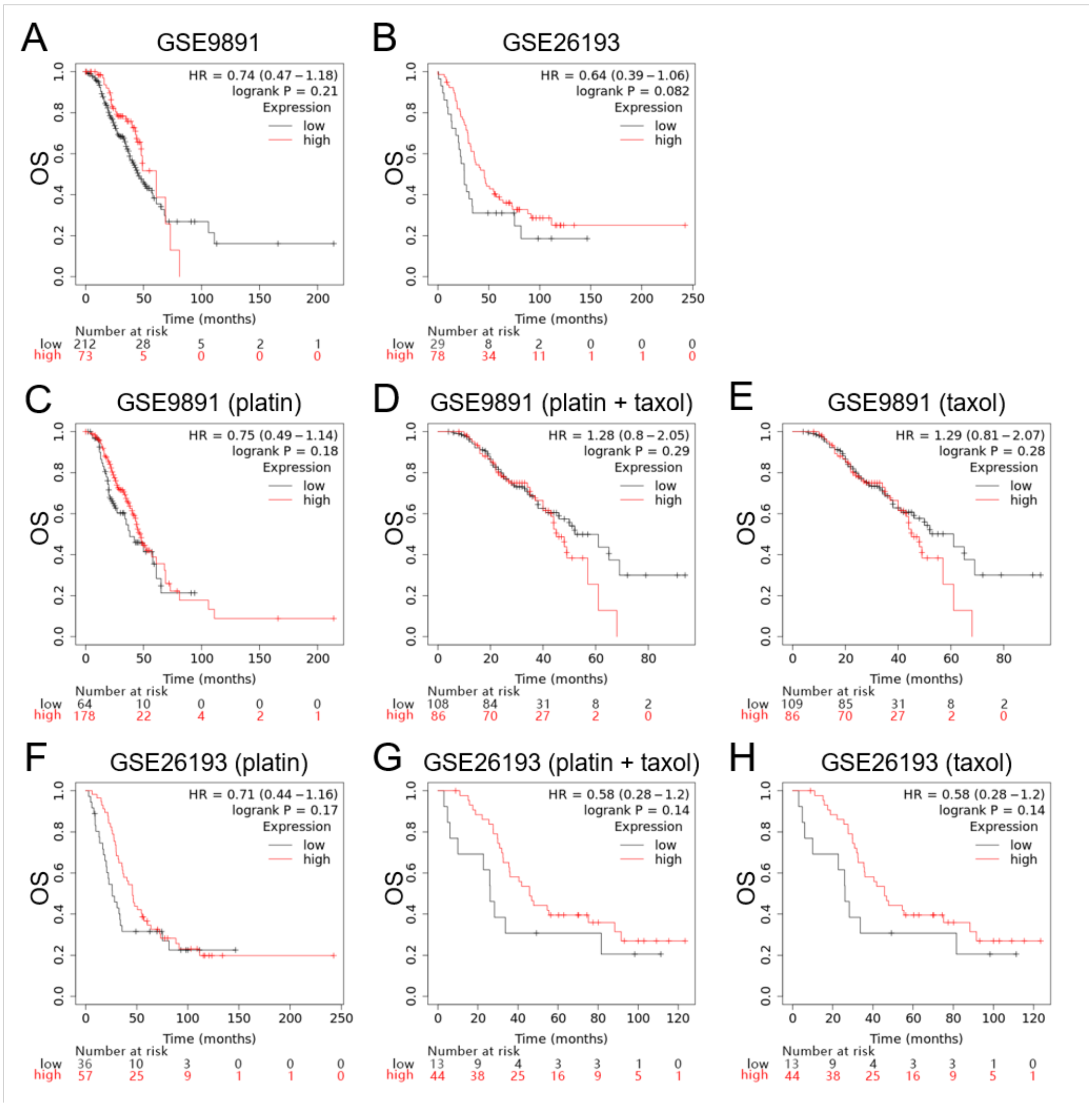

Supplementary Figure S3. Kaplan-Meier curves based on MAPK15 expression showing overall survival (OS) of patients with ovarian carcinoma. (**A**) OS according to GSE9891. (**B**) OS according to GSE26193. (**C**–**E**) OS according to GSE9891 with platin and/or taxol chemotherapy. (**F**–**H**) OS according to GSE26193 with platin and/or taxol chemotherapy.
